# The effect of different starter cultures on biogenic amines and quality of fermented mutton sausages stored at 4 and 20°C temperatures

**DOI:** 10.1002/fsn3.1748

**Published:** 2020-07-08

**Authors:** Xueying Sun, Bao Du, Lihua Zhao, Ye Jin, Lin Su, Jianjun Tian, Jing Wu

**Affiliations:** ^1^ College of Food Science and Engineering Inner Mongolia Agricultural University Hohhot People's Republic of China

**Keywords:** biogenic amines, fermented mutton sausage, starter culture, storage

## Abstract

The biogenic amines (BAs), water activity, pH, thiobarbituric acid‐reactive substances (TBARS), and nitrite were, respectively, tested in dry fermented sausage with starter cultures (37x‐6 *Lactobacillus plantarum*, x3‐3b *L. plantarum*, 30x‐11 *Staphylococcus* *pentosans*, and 37x‐8 *S*. *pentosans*), during storage of room temperature (20°C) and refrigeration storage (4°C). Tryptamine (TRM), 2‐phenylethylamine (PHE), putrescine (PUT), cadaverine (CAD), histamine (HIM), and tyramine (TYM) contents of all samples were increased storage at 20°C, and the content of TRM, PUT, CAD, and HIM of all samples storage at 20°C was higher than that storage at 4°C after 42 days. The content of BA with 37x‐6, x3‐3b, and 37x‐8 was obviously decreased at 4°C storage. The storage temperature has a significant effect on BA content (*p* < .05) for TYM and other BAs tested. Finally, x3‐3b, 37x‐6, and 37x‐8 should be used to produce fermented sausages on the basis of the concentration of BAs.

## Graphical abstract

In summary, the effect of the storage temperature and starter culture on BAs content was significant, the starter culture of x3‐3b, 37x‐6, and 37x‐8 and low temperature storage would be optimum for producing and storing fermented sausage regarding biogenic amine concentrations.

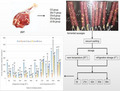

## 1 INTRODUCTION

Biogenic amines (BAs) are low molecular organic bases with nitrogen synthesized in the metabolism of microorganisms, plants, and animals (Kamil & Khalid, 2018). The most important BAs detected are tryptamine (TRM), 2‐phenylethylamine (PHE), putrescine (PUT), cadaverine (CAD), histamine (HIM), tyramine (TYM), spermidine (SPD), and spermine (SPM), and they are synthesized by decarboxylation of counterpart amino acids with the exception of SPD and SPM (Zhang, Lin, & Nie, 2013). The low concentrations of BAs are essential for many physiological functions (Önal, Tekkeli, & Önal, 2013). However, toxicity will be produced when BAs accumulate to a high amount in the human body. It can cause headaches, nausea, heart disease, and other typical symptoms (Shalaby, 1996). HIM is the most toxic BA. In addition to its own effects, the presence of other BAs will increase their toxicity. In addition, CAD and PUT can form carcinogenic nitrosamines (Önal, 2007; Teti, Visalli, & Mcnair, 2002). Biogenic amines exist not only in various animal and plant tissues, but also in fermented foods with rich protein content (Lu et al., 2010; Sun et al., 2016; Tang et al., 2011).

Fermented sausage produced from meat and fat particles, salt, spices, and condiments with starter culture fermentation can be stored for long time due to its low water activity (0.7–0.8) and low pH (4.5–5.5) (Ana, Rodriguez, & Hidalgo, 2016; Manuela, Juan, José, Herranz, & Hoz, 2000). Many researchers found content of BAs in a great deal of different types of fermented sausages. Latorre‐Moratalla et al found that the main BAs were tyramine, PUT, and CAD, with the highest content of CAD of 621 mg/kg, and tyramine and PUT of 473 and 449 mg/kg in European traditional fermented sausage (2008) Hüseyin, Güzin,& Mükerrem, 2007); therefore, the choice of starter cultures is significant for the formation of BAs. Aymerich, Garriga, Monfort, Nes, and Hugas (2000) found that inoculated of CTC 494 (*Lactobacilli sake*) in sausages can reduce BAs during storage, especially TYM, PUT, and CAD.

In the study of the effect of storage temperature on BAs, there are relatively few reports and different opinions. Komprda, Smělá, Pechová, Kalhotka, and Klejdus (2004) investigated that the storage temperature had an effect on the content of BAs. The content of BAs was higher at room temperature (20°C) than at refrigeration storage (4°C). When the temperature reached about 4°C, the species and quantity of aminobacteria decreased, and the activities of proteolytic enzyme and amino acid decarboxylase decreased. However, Komprda, Neznalovà, Standara, and Bovercid (2001) found that the content of BAs was not significantly different between sausages stored at 8 and 22°C for 60 days. The influence of temperature on the content of BAs during the storage of fermented sausage needs to be continued to study.

The aim of the present work was to study the effect of different starter culture of 30x‐11 *Staphylococcus pentosans*, 37x‐6 *Lactobacillus plantarum*, 37x‐8 *S. pentosans*, and x3‐3b *L. plantarum* on BA accumulation at refrigeration storage (4°C) and at room temperature (20°C) during storage of fermented mutton sausage. Other parameters (pH, water activity, TBARS, and nitrite) that might provide further information on the product under study were also determined.

## 2 MATERIALS AND METHODS

### 2.1 MATERIALS

The materials used including raw meat from the hindquarter of sheep and fat from the tail of sheep in this research obtained from Bayannur City (Inner Mongolia, China) were directly transferred to Meat Laboratory (College of Food Science and Engineering, Inner Mongolia Agricultural University, Inner Mongolia, China). Standard amines, containing TRY, PHE, PUT, CAD, HIS, SPD, and TYR, were purchased from Sigma, acetonitrile, acetone, n‐hexane, acetic acid and ammonium acetate (all of them HPLC grade), other chemicals used were of analytical grade. 30x‐11 *S. pentosans*, 37x‐6 *L. plantarum*, 37x‐8 *S. pentosans*, *and* x3‐3b *L. plantarum* were isolated from fermented beef jerky from Mongolian and fermented sausage from Meat Laboratory (College of Food Science and Engineering, Inner Mongolia Agricultural University, Inner Mongolia, China). All of the meat and fat were weighed in advance and cut. Five different sample groups of fermented mutton sausages were produced with various of starter cultures. (a) CO: without starter culture; (b) 30x‐11: with 30x‐11 *Pentose Staphylococcus*; (c) 37x‐6: with 37x‐6 *L. plantarum*; (d) 37x‐8: with 37x‐8 *Staphylococcus pentosae*; and (e) x3‐3b: with x3‐3b *L. plantarum*.

The sausage was prepared according to the following formulation lean mutton meat (90% w/w), fat (10% w/w), sucrose (0.5% w/w), sodium nitrate (0.01% w/w), trite (0.007% w/w) glucose (0.5% w/w), and starter (2% w/w) (Zhao et al., 2011). Mutton meat, fat, and other ingredients were mixed with starter cultures, which were made into 26 mm particle size in freedom regimen. The mixed was pickled for 12 hr at 4°C and then packed into the natural goat sausage coat and ligated with the length of 10 cm. After exhausted and rinsed, the sausage was suspended and fermented at 24.5°C and 95% relative humidity (RH) for 2 days in constant temperature and humidity culture. Then separately at the condition of 14.5°C, 90% RH, and 14.5°C, 80% RH entered drying and ripening process. Then, the sausage was matured for 2 days at 14.5°C and 90% relative humidity, vacuum packed and storage at room temperature (20°C) and low temperature (4°C). Samples were obtained at 0, 21, 42, 63, and 84 days and were taken for subsequent analysis of the experiment.

### 2.2 METHODS

#### 2.2.1 Determination of pH and a_w_

The pH values of fermented sausages were tested making use of a pH meter (Model 400, Alliance Instrument Technology Co., Ltd) that was calibrated with 4.01, 6.86, and 9.18 pH solutions. 36 ml of distilled water was added to 4 g of each sample, stirred for 30 min, and pH values were measured. Water activity was determined with a moisture activity meter (model CH8853; Novasina).

#### 2.2.2 Determination of color

Colors of fermented sausages were tested using a Chroma Meter HPC‐226 (Oek Instruments Co., Ltd) which was standardized with a white plate. Color was showed as L* (brightness/darkness), a* (greenness/redness), b* (blueness/yellowness), and e (chromatic aberration parameter).

#### 2.2.3 Determination of TBARS

The content of TBARS was determined to assess the lipid oxidation levels in fermented sausages storage at different temperatures, using the method of Sinnhuber and Yu (1977). Simply, 20 ml of 4% trichloroacetic acid (containing 0.1% EDTA) was added to 5 g of each chopped sample, shaked for 30 min, and then filtered. The filtrate (5 ml per sample) was transferred to a separate tube and mixed with 5 ml of 0.02 mol/L TBAR solution, then heated with the extraction temperature 90°C for 40 min, put it in cold water 1 hr and centrifuged for 5 min, about 8 ml of supernatant, respectively, was taken, and 5 ml of trichloromethane was added, swirled for 2 min, and kept for 15–20 min. The absorbance was measured at 532 and 600 nm using an UV‐visible spectrophotometer (UV‐1800; MAPADA).

#### 2.2.4 Determination of nitrite

The content of nitrite was tested to assess the safety of fermented sausages storage at different temperatures, using the method of GB 5009.33–2010 of China (2016). Briefly, 12.5 ml of 50 g/L saturated borax solution was added to 5 g of each chopped sample, added 150 ml of 70°C water, heated in boiling water bath for 15min. 5 ml of 106 g/L potassium ferrocyanide solution and 5 ml of 220 g/L zinc acetate solution were added to precipitate protein. Then, 40.0 ml of filtrate, 2 ml of 4 g/L p‐aminobenzenesulfonic acid solution, and 1 ml of 2 g/L naphthalene ethylenediamine hydrochloride solution were mixed, and water was added and allowed to stand for 15 min. The absorbance was measured at 538 nm using an UV‐visible spectrophotometer (UV‐1800; MAPADA).

#### 2.2.5 Determination of biogenic amines

The content of BAs in sausages was determined with reference to GB 5009.208–2016 of China (2016). Five gram of sample was extracted with 5% trichloroacetic acid, removed fat by hexane, purified by the mixed solution of chloroform and n‐butanol (1:1), and then derivatized by dansulfonyl chloride. After derivatization, filtered with 0.22 μm aperture filter, 10 µl filtrate of each sausage was injected onto a chromatographic column (ZORBAX SB‐C18: 250 mm × 4.6 mm, 5 µl particle size, Agilent) connected to a high‐performance liquid chromatography (HPLC; Agilent 1260). Filtrate was separated by gradient elution with mobile phases A (acetonitrile and ammonium acetate with 0.1% acetic acid [9:1]) and mobile phases B (acetonitrile and ammonium acetate with 0.1% acetic acid(1/9)) (time 0–22 min: mobile phases A, 60%–85%, mobile phases B, 40%–15%; 25–32 min: mobile phases A, 100% and 32.01–37 min: mobile phases A, 60%, mobile phases B, 40%), at a flow rate of 0.8 ml/min using the UV detection wavelength at 254 nm. Compared with the retention times of known standards, the separated amines were identified.

#### 2.2.6 Statistical analysis

Excel and SPSS 18.0 software were used for data statistics and significant difference analysis. The obtained data were analyzed by the general linear model procedure considering treatment as the main effect. Means were compared using Duncan's multiple range test, with a significance of *p* < .05. Correlations between variables were determined by correlation analyses using the Pearson linear correlation coefficient with the above statistical software package.

## 3 RESULTS AND DISCUSSION

### 3.1 Water activity and pH

The results for a_w_ and pH determinations are reported during storage of all samples at four starter cultures and two storage temperatures are listed in Table 1. The decrease of a_w_ values was small and the low of change was not obvious. The free degree of water in sausage was high and the combination degree with sausage was low storage at 4°C, so the a_w_ values were higher than storage at 20°C, which indicated that the storage temperature had a big impact on the a_w_ values of fermented mutton sausage.

**TABLE 1:** Changes in pH value and water activity of fermented mutton sausages

	Sample	Temperature of storage (°C)	Time of storage (days)				
			0	21	42	63	84
pH	CO	4	5.90 ± 0.14bB	5.76 ± 0.00aB	5.85 ± 0.01bB	5.53 ± 0.06bA	5.47 ± 0.01bA
	x3‐3b		5.09 ± 0.12aB	5.14 ± 0.01bB	5.47 ± 0.12aC	4.89 ± 0.16aB	4.61 ± 0.03aA
	30x‐11		5.96 ± 0.57bA	5.67 ± 0.02cA	6.22 ± 0.02cA	5.64 ± 0.23bA	5.58 ± 0.15cA
	37x‐6		5.63 ± 0.28abA	6.00 ± 0.00dA	5.97 ± 0.09bA	5.69 ± 0.19bA	5.64 ± 0.07dA
	37x‐8		5.95 ± 0.07bA	5.87 ± 0.02eA	6.40 ± 0.03cB	5.69 ± 0.33bA	5.60 ± 0.11cA
	CO	20	5.90 ± 0.14bBC	5.53 ± 0.10bA	6.06 ± 0.08bC	5.69 ± 0.00aAB	5.62 ± 0.01aA
	x3‐3b		5.09 ± 0.12aBC	4.91 ± 0.01aA	5.24 ± 0.03aC	4.94 ± 0.01bAB	5.69 ± 0.01aD
	30x‐11		5.96 ± 0.57bAB	5.52 ± 0.05bA	6.58 ± 0.03dB	6.09 ± 0.03cAB	5.95 ± 0.14bAB
	37x‐6		5.63 ± 0.28abA	5.62 ± 0.08bA	6.28 ± 0.08cB	5.48 ± 0.04dA	6.02 ± 0.02bB
	37x‐8		5.95 ± 0.07bAB	5.81 ± 0.01cAB	6.60 ± 0.00dC	6.00 ± 0.04eB	5.80 ± 0.14abA
Water activity	CO	4	0.858 ± 0.001bcC	0.846 ± 0.001bBC	0.834 ± 0.013aAB	0.821 ± 0.001aA	0.845 ± 0.002aBC
	x3‐3b		0.811 ± 0.001aA	0.821 ± 0.003aA	0.850 ± 0.001abAB	0.855 ± 0.002cAB	0.881 ± 0.055aB
	30x‐11		0.850 ± 0.004bB	0.858 ± 0.004bB	0.873 ± 0.004cC	0.832 ± 0.006abA	0.874 ± 0.014aC
	37x‐6		0.856 ± 0.006bcA	0.875 ± 0.014cB	0.874 ± 0.007cAB	0.855 ± 0.010cA	0.870 ± 0.016aAB
	37x‐8		0.864 ± 0.000cB	0.842 ± 0.001bA	0.871 ± 0.011bcB	0.849 ± 0.005bcA	0.847 ± 0.002aA
	CO	20	0.858 ± 0.001bcD	0.844 ± 0.004bD	0.826 ± 0.014aC	0.769 ± 0.003aA	0.798 ± 0.003aA
	x3‐3b		0.811 ± 0.001aA	0.861 ± 0.014bC	0.832 ± 0.001aB	0.824 ± 0.004cAB	0.831 ± 0.001dB
	30x‐11		0.850 ± 0.004bB	0.813 ± 0.010aA	0.843 ± 0.004abB	0.836 ± 0.001dB	0.817 ± 0.004bA
	37x‐6		0.856 ± 0.006bcC	0.861 ± 0.006bC	0.854 ± 0.006bC	0.787 ± 0.007bA	0.839 ± 0.003dB
	37x‐8		0.864 ± 0.000cC	0.835 ± 0.011abB	0.833 ± 0.015aB	0.797 ± 0.023bA	0.823 ± 0.007bcB

Note:

All values are given in mean ± *SD*, where *n* = 3. Values followed by the same capital letters (A–E) in a row and lowercase letters (a–e) in a column are not statistically different (*P < *.05). CO: control without starter culture; 30x‐11: with 30x‐11 *Pentose Staphylococcus*; 37x‐6: with 37x‐6 *Lactobacillus plantarum*; 37x‐8 : with 37x‐8 *Staphylococcus pentosae*; x3‐3b: with x3‐3b *Lactobacillus plantarum*.

pH value is a key consideration affecting BA formation; especially, pH value between 4.0 and 5.5 is the optimum condition for the enzyme to form BAs (Sadiye & Özgül, 2019; Santos, 1996). pH values varied from 4.61 to 6.60 during the storage of fermented sausages. Significant correlations were found between the acidities, and PHE (*r* = .337, *p* < .05), PUT (*r* = .367, *p* < .05), CAD (*r* = .408, *p* < .05), HIM (*r* = .306, *p* < .05), SPD (*r* = −.321, *p* < .05), and total amine contents (*r* = .379, *p* < .05). The pH values of sausage stored at 20°C are slightly lower than that sausage stored at 4°C at 21th day of storage. This pattern of pH values was similar with the study on sausage production of Peng, Wu, Jia, and Tang (1999). The reason may be that the high temperature can enhance the activity of various enzymes, accelerate the change of components in fermented sausage, and promote the glycolysis in fermented sausage. A rise of pH was related to the decomposition of lactic acid following the depletion of the sugar (Klettner & List, 1978; Komprda et al., 2001). The pH value of sausages began to rise after 42 days of storage and stored at 20°C was basically higher than that of sausage stored at 4°C in our study. An increase in pH was possibly connected with protein hydrolytic activity of the starter culture to form peptides, amino acids, and ammonia (Komprda et al., 2001).

### 3.2 Color values

The color values of fermented sausages are shown in Table 2. L* values of all experimental groups of fermented sausages samples were significantly more than that in the control group (*p* < .05) from the 21th day to the 63th of storage at 4°C. In the whole storage process, the a* value of the experimental group was always greater than that sample CO, and there was a significant difference between the four groups of sausages (*p* < .05), which indicated that addition of starter cultures could effectively improve the a* value of fermented sausages. A different trend was noted for b* value in the whole storage process, which indicate that starter cultures have no significant effect on b* value in color difference (*p* > .05).

**TABLE 2:** Changes in the color of fermented mutton sausages

	Sample	Temperature of storage (°C)	Time of storage (days)				
			0	21	42	63	84
L^*^	CO	4	48.85 ± 1.02^bC^	43.09 ± 0.42^bB^	37.19 ± 0.51^aA^	37.81 ± 1.48^aA^	43.43 ± 2.02^bB^
	x3‐3b		46.80 ± 2.62^abB^	44.12 ± 0.11^cB^	39.87 ± 1.63^bA^	44.56 ± 1.95^cB^	45.83 ± 0.77^cB^
	30x‐11		43.40 ± 1.37^aB^	43.10 ± 0.79^bB^	43.30 ± 0.35^cB^	40.73 ± 0.38^bA^	42.91 ± 0.18^abB^
	37x‐6		44.25 ± 2.64^aBC^	44.92 ± 0.28^aC^	43.63 ± 0.96^cABC^	42.12 ± 1.34^bAB^	41.26 ± 0.67^aA^
	37x‐8		46.02 ± 0.76^abD^	38.12 ± 0.08^aA^	44.1 ± 1.28^cC^	41.30 ± 0.71^bB^	43.14 ± 0.39^abC^
	CO	20	48.85 ± 1.02^bC^	42.72 ± 0.42^bAB^	39.31 ± 3.79^abA^	40.01 ± 0.04^aA^	44.92 ± 1.51^aB^
	x3‐3b		46.80 ± 2.62^abA^	44.56 ± 0.46^cA^	45.52 ± 0.31^cA^	43.35 ± 0.66^bA^	46.24 ± 3.94^aA^
	30x‐11		43.40 ± 1.37^aA^	40.47 ± 0.03^aB^	38.34 ± 1.13^aC^	50.23 ± 1.49^cD^	45.77 ± 0.32^aE^
	37x‐6		44.25 ± 2.64^aA^	45.40 ± 0.33^dA^	46.67 ± 1.39^cAB^	45.82 ± 0.66^dA^	48.75 ± 0.98^aB^
	37x‐8		46.02 ± 0.76^abB^	42.24 ± 0.38^bA^	43.02 ± 1.91^bcA^	48.22 ± 0.15^eC^	48.04 ± 0.83^aC^
a*	CO	4	10.34 ± 0.63^aA^	16.25 ± 0.27^aB^	17.38 ± 0.27^aB^	16.83 ± 0.30^aB^	18.94 ± 1.10^aC^
	x3‐3b		16.71 ± 0.64^bA^	17.58 ± 0.05^bA^	21.06 ± 0.51^bC^	18.91 ± 1.09^bB^	21.55 ± 0.62^bC^
	30x‐11		19.19 ± 0.55^cA^	19.18 ± 0.99^cA^	21.66 ± 0.36^bB^	19.68 ± 0.28^bA^	22.08 ± 0.15^bB^
	37x‐6		19.27 ± 3.28^bAB^	20.04 ± 0.26^cAB^	20.91 ± 0.86^bAB^	18.84 ± 1.28^bA^	22.15 ± 0.40^bB^
	37x‐8		17.27 ± 0.41^bA^	20.92 ± 0.02^dC^	21.11 ± 1.14^bC^	19.81 ± 1.16^bBC^	19.40 ± 0.44^aB^
	CO	20	10.34 ± 0.63^aA^	17.28 ± 0.64^aBC^	19.71 ± 2.50^aC^	17.19 ± 0.12^aB^	17.97 ± 1.03^aBC^
	x3‐3b		16.71 ± 0.64^bA^	20.50 ± 0.17^cB^	20.52 ± 1.03^aB^	20.77 ± 0.32^cB^	20.05 ± 0.20^cB^
	30x‐11		19.19 ± 0.55^cA^	19.36 ± 0.55^bA^	23.68 ± 0.09^bB^	19.41 ± 1.52^bcA^	19.72 ± 0.10^bcA^
	37x‐6		19.27 ± 3.28^bA^	20.56 ± 0.12^cA^	20.35 ± 1.36^aA^	18.71 ± 0.36^bA^	20.22 ± 0.57^cA^
	37x‐8		17.27 ± 0.41^bA^	19.77 ± 0.43^bcB^	21.14 ± 1.11^abC^	19.62 ± 0.20^bcB^	18.91 ± 0.39^abB^
b^*^	CO	4	7.46 ± 0.43^abC^	5.7 ± 0.04^aA^	6.75 ± 0.17^aB^	5.68 ± 0.12^aA^	8.53 ± 0.62^abD^
	x3‐3b		6.35 ± 0.23^aA^	5.95 ± 0.08^abA^	7.81 ± 0.06^bBC^	7.04 ± 1.33^aAB^	8.29 ± 0.07^abC^
	30x‐11		7.80 ± 0.53^abB^	6.08 ± 0.55^abA^	7.92 ± 0.14^bB^	5.81 ± 0.10^aA^	8.78 ± 0.16^bC^
	37x‐6		8.28 ± 1.63^bB^	7.58 ± 0.05^dAB^	8.93 ± 0.22^cB^	6.42 ± 0.72^aA^	8.64 ± 0.07^bB^
	37x‐8		7.42 ± 0.16^abB^	6.42 ± 0.17^cA^	8.35 ± 0.10^dB^	6.31 ± 0.37^aA^	7.46 ± 1.10^aB^
	CO	20	7.46 ± 0.43^abB^	6.62 ± 0.23^aA^	8.30 ± 0.19^abC^	6.38 ± 0.14^aA^	10.22 ± 0.59^bD^
	x3‐3b		6.35 ± 0.23^aA^	7.12 ± 0.21^aB^	8.88 ± 0.29^cC^	7.51 ± 0.06^bB^	9.24 ± 0.40^aC^
	30x‐11		7.80 ± 0.53^abB^	6.07 ± 0.13^aA^	8.96 ± 0.17^cC^	8.33 ± 0.46^cBC^	8.66 ± 0.13^aC^
	37x‐6		8.28 ± 1.63^bAB^	6.40 ± 1.69^aA^	8.56 ± 0.52^bcB^	7.24 ± 0.34^bAB^	8.97 ± 0.19^aB^
	37x‐8		7.42 ± 0.16^aB^	6.68 ± 0.07^aE^	7.96 ± 0.15^aC^	8.70 ± 0.10^cD^	9.25 ± 0.46^aE^
e	CO	4	1.60 ± 0.02^aA^	3.22 ± 0.04^aB^	3.04 ± 0.04^bC^	3.41 ± 0.09^abD^	2.66 ± 0.12^aE^
	x3‐3b		2.99 ± 0.17^bA^	3.35 ± 0.03^bA^	3.22 ± 0.11^cA^	3.18 ± 0.57^aA^	3.10 ± 0.10^bA^
	30x‐11		2.91 ± 0.26^bA^	3.61 ± 0.09^cC^	3.24 ± 0.01^cB^	3.87 ± 0.00^bD^	3.03 ± 0.04^bAB^
	37x‐6		2.77 ± 0.26^bA^	3.09 ± 0.02^dB^	2.82 ± 0.07^aA^	3.40 ± 0.15^abB^	3.10 ± 0.04^bB^
	37x‐8		2.70 ± 0.05^bA^	3.80 ± 0.09^eC^	3.01 ± 0.14^bAB^	3.62 ± 0.06^abC^	3.09 ± 0.37^bB^
	CO	20	1.60 ± 0.02^aA^	3.01 ± 0.03^aC^	2.88 ± 0.41^aC^	3.13 ± 0.04^cC^	2.16 ± 0.11^aB^
	x3‐3b		2.99 ± 0.17^bB^	3.34 ± 0.06^abC^	2.76 ± 0.08^aA^	3.24 ± 0.04^dC^	2.61 ± 0.16^bcA^
	30x‐11		2.91 ± 0.26^bA^	3.67 ± 0.03^abC^	3.26 ± 0.08^aB^	2.71 ± 0.10^aA^	2.71 ± 0.05^cA^
	37x‐6		2.77 ± 0.26^bA^	3.82 ± 0.85^bB^	2.83 ± 0.33^aA^	3.00 ± 0.07^bA^	2.67 ± 0.11^bcA^
	37x‐8		2.70 ± 0.05^bB^	3.42 ± 0.04^abD^	3.15 ± 0.16^aC^	2.66 ± 0.02^aB^	2.44 ± 0.16^bA^

Note:

All values are given in mean ± *SD*, where *n* = 3. Values followed by the same capital letters (A–E) in a row and lowercase letters (a–e) in a column are not statistically different (*P < *.05). CO: control without starter culture; 30x‐11: with 30x‐11 *Pentose Staphylococcus*; 37x‐6: with 37x‐6 *Lactobacillus plantarum*; 37x‐8: with 37x‐8 *Staphylococcus pentosae*; x3‐3b: with x3‐3b *Lactobacillus plantarum*.

The change trend of color of sausage stored at two temperatures was the same, e value increased from 0 to 21 days, decreased first, then increased from 21 to 63 days, and e value began to decrease again from 63 to 84 days. The e value of fermented sausage stored at 4 and at 20°C was significantly greater than that of sample CO at 84th day (*p* < .05). It showed that the starter can effectively improve the color of fermented mutton sausages, which is the same as the research results of Lorenzo, Gómez, and Fonseca (2014).

### 3.3 TBARS of sausages samples

TBARS is the most widely used way to express the lipid oxidation degree of meat products. The high TBARS value indicates that the degree of lipid oxidation is high and the quality of meat products is poor. Generally speaking, the reason of sausage deterioration is not only the growth of spoilage microorganisms, but also the lipid oxidation. During the storage of sausage, the lipid oxidation will produce some organic compounds with pungent smell, which will make it produce peculiar smell, and reduce the product quality and sales volume. In the process of lipid oxidation, some peroxides will be produced, which are harmful to human health (Guo et al., 2015). During the whole storage period, the TBARS values of fermented sausages stored at 4°C were lower than that of sausage stored at 20°C. This indicated that the lipid oxidation degree was small and TBARS values were low at low temperature. This is consistent with the conclusion of Wang (2013). With the increase in temperature, the change range of TBARS value increases, and there was a positive correlation between the degree of lipid oxidation in fermented sausage and storage temperature (Table 3).

**TABLE 3:** Changes in TBARS values (mg/100 g) of fermented mutton sausages

Sample	Temperature of storage (°C)	Time of storage (days)				
		0	21	42	63	84
CO	4	0.341 ± 0.001^eC^	0.304 ± 0.007cB	0.459 ± 0.020cD	0.239 ± 0.007dA	0.351 ± 0.012bC
x3‐3b		0.220 ± 0.003^dA^	0.262 ± 0.012bB	0.248 ± 0.011bB	0.211 ± 0.000cA	0.192 ± 0.000cA
30x‐11		0.153 ± 0.010^aB^	0.187 ± 0.001aB	0.300 ± 0.015aC	0.108 ± 0.001aA	0.145 ± 0.006aB
37x‐6		0.171 ± 0.001^bB^	0.178 ± 0.002aB	0.239 ± 0.001dC	0.150 ± 0.002bA	0.155 ± 0.001aA
37x‐8		0.192 ± 0.000^cB^	0.197 ± 0.000aC	0.215 ± 0.003eD	0.150 ± 0.006bA	0.192 ± 0.003bB
CO	20	0.341 ± 0.001^eA^	0.407 ± 0.002dB	0.520 ± 0.019cC	0.422 ± 0.015cB	0.361 ± 0.008cA
x3‐3b		0.220 ± 0.003^dA^	0.384 ± 0.011cC	0.511 ± 0.009cD	0.356 ± 0.007bB	0.351 ± 0.012bB
30x‐11		0.153 ± 0.010^aA^	0.169 ± 0.005aA	0.286 ± 0.005aB	0.300 ± 0.003bB	0.319 ± 0.001aB
37x‐6		0.171 ± 0.001^bA^	0.248 ± 0.002bB	0.389 ± 0.014bC	0.281 ± 0.009aD	0.320 ± 0.003aE
37x‐8		0.192 ± 0.000^cA^	0.225 ± 0.001bB	0.328 ± 0.011bC	0.276 ± 0.010aD	0.351 ± 0.008bE

Note:

All values are given in mean ± *SD*, where *n* = 3. Values followed by the same capital letters (A–E) in a row and lowercase letters (a–e) in a column are not statistically different (*P < *.05). CO: control without starter culture; 30x‐11: with 30x‐11 *Pentose Staphylococcus*; 37x‐6: with 37x‐6 *Lactobacillus plantarum*; 37x‐8 : with 37x‐8 *Staphylococcus pentosae*; x3‐3b: with x3‐3b *Lactobacillus plantarum*.

### 3.4 Nitrite of sausages samples

As shown in Table 4, the nitrite residue of sausage stored at 4°C and at 20°C changes significantly. Throughout the whole storage stage, the nitrite content of sausage stored at 4°C was higher than that stored at 20°C compare the same group at the same time, which showed that high storage temperature leads to low nitrite content of sausages in a certain range, which is consistent with the research results of Jia, Tang, Peng, and Wu (2001). The reason may be that high storage temperature promotes a series of biochemical changes in meat, enhances the binding ability of nitrite and myoglobin, and reduces the nitrite residue in sausages.

**TABLE 4:** Variation of nitrite content (mg/kg) in fermented mutton sausages

Sample	Temperature of storage (°C)	Time of storage (days)				
		0	21	42	63	84
CO	4	19.99 ± 0.33^cA^	8.14 ± 0.25^bB^	4.42 ± 0.01^aC^	15.56 ± 0.44^cD^	5.38 ± 0.28^bE^
x3‐3b		16.09 ± 0.33^aB^	7.15 ± 0.41^aC^	4.24 ± 0.04^aA^	14.24 ± 0.48^bD^	4.12 ± 0.30^aA^
30x‐11		16.54 ± 0.82^abC^	7.40 ± 0.10^aA^	7.65 ± 0.08^cA^	13.90 ± 0.31^abB^	6.93 ± 0.25^cA^
37x‐6		17.43 ± 0.66^abD^	8.56 ± 0.07^bB^	7.61 ± 0.18^cA^	13.12 ± 0.37^aC^	6.88 ± 0.24^cA^
37x‐8		17.68 ± 0.59^bA^	8.18 ± 0.14^bB^	5.82 ± 0.07^bC^	15.74 ± 0.21^cD^	6.90 ± 0.28^cE^
CO	20	19.99 ± 0.33^cD^	6.23 ± 0.28^dB^	6.59 ± 0.48^dB^	11.71 ± 0.33^cC^	5.08 ± 0.06^bA^
x3‐3b		16.09 ± 0.33^aE^	3.32 ± 0.30^aB^	2.59 ± 0.01^aA^	8.38 ± 0.42^aD^	4.42 ± 0.11^aC^
30x‐11		16.54 ± 0.82^abC^	5.27 ± 0.24^cA^	5.18 ± 0.31^cA^	9.19 ± 0.37^abD^	5.07 ± 0.10^bA^
37x‐6		17.43 ± 0.66^abC^	4.26 ± 0.24^bA^	4.37 ± 0.16^bA^	9.80 ± 0.41^bB^	4.50 ± 0.03^aA^
37x‐8		17.68 ± 0.59^bD^	4.91 ± 0.27^bcA^	4.62 ± 0.13^bcA^	13.99 ± 0.49^dC^	6.68 ± 0.14^cB^

Note:

All values are given in mean ± *SD*, where *n* = 3. Values followed by the same capital letters (A–E) in a row and lowercase letters (a–e) in a column are not statistically different (*P < *.05). CO: control without starter culture; 30x‐11: with 30x‐11 *Pentose Staphylococcus*; 37x‐6: with 37x‐6 *Lactobacillus plantarum*; 37x‐8 : with 37x‐8 *Staphylococcus pentosae*; x3‐3b: with x3‐3b *Lactobacillus plantarum*.

### 3.5 BA contents during storage

**FIGURE 1:**
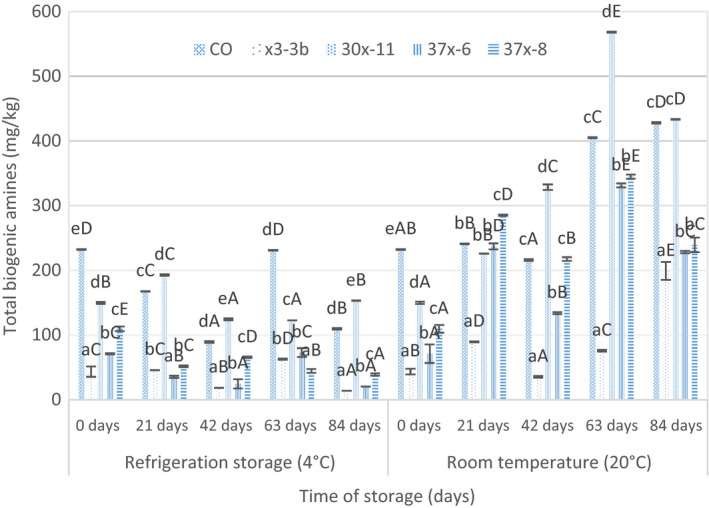
The total biogenic amine contents (mg/kg) of samples during the storage. Note: Values followed by the same capital letters (A–E) in a row and lowercase letters (a–e) in a column are not statistically different (*P < *.05). CO: control without starter culture; 30x‐11: with 30x‐11 *Pentose Staphylococcus*; 37x‐6: with 37x‐6 *Lactobacillus plantarum*; 37x‐8 : with 37x‐8 *Staphylococcus pentosae*; x3‐3b: with x3‐3b *Lactobacillus plantarum*

From Table 5, it can be seen that the changes in BAs during storage of fermented sausages. The contents of the seven BAs were very low (especially TRM) and remained unchanged at the beginning of storage in sausage samples. BAs began to raise at the later stage of storage. Figure 1 shows that the activity of decarboxylase x3‐3b, 37x‐6, and 37x‐8 was negative and 30x‐11 was positive during the storage period of fermented sausage at 4 and 20°C. With the prolongation of storage time, the content of BA increased continuously stored at 20°C and higher than the content of BAs stored at 4°C.

**TABLE 5:** Biogenic amine contents (mg/kg) of samples during the storage

	Sample	Temperature of storage (°C)	Time of storage (days)				
			0	21	42	63	84
Tyramine	CO	4	141.70 ± 0.71^dD^	133.48 ± 6.20^dC^	63.92 ± 1.42^dA^	178.25 ± 1.24^dE^	83.97 ± 2.76^cB^
	x3‐3b		20.70 ± 0.20^aC^	39.37 ± 0.25^cD^	15.82 ± 1.13^bB^	54.53 ± 1.22^bE^	12.19 ± 1.22^aA^
	30x‐11		77.51 ± 1.41^cA^	153.78 ± 0.01^eD^	111.32 ± 1.41^eB^	112.90 ± 6.29^cB^	124.61 ± 0.99^dC^
	37x‐6		23.53 ± 0.71^aC^	15.88 ± 0.41^aB^	8.02 ± 0.01^aA^	35.09 ± 2.11^aD^	10.96 ± 2.48^aA^
	37x‐8		53.94 ± 1.27^bC^	26.86 ± 0.05^bB^	19.23 ± 0.28^cA^	28.90 ± 0.27^aB^	20.47 ± 1.40^bA^
	CO	20	141.70 ± 0.71^dB^	145.51 ± 3.61^dB^	79.01 ± 2.83^cA^	202.13 ± 14.47^dC^	229.88 ± 5.64^cD^
	x3‐3b		20.70 ± 0.20^aA^	40.83 ± 0.18^aB^	26.33 ± 0.42^aC^	55.49 ± 3.04^aD^	64.74 ± 1.07^aE^
	30x‐11		77.51 ± 1.41^cA^	114.00 ± 0.44^cB^	138.96 ± 6.36^dC^	169.07 ± 13.7^cE^	180.26 ± 1.74^bD^
	37x‐6		23.53 ± 0.71^aA^	68.35 ± 1.24^bB^	69.81 ± 0.71^bBC^	120.52 ± 2.23^bD^	71.03 ± 1.07^aC^
	37x‐8		53.94 ± 1.27^bA^	57.26 ± 1.46^bAB^	66.86 ± 0.08^bAB^	128.17 ± 0.31^bC^	78.11 ± 10.87^aB^
Histamine	CO	4	5.45 ± 0.07^aA^	11.52 ± 0.71^cB^	14.91 ± 0.00^aC^	26.28 ± 0.18^cD^	12.44 ± 0.49^cB^
	x3‐3b		ND	ND	0.72 ± 0.01^bA^	0.45 ± 0.04^aB^	ND
	30x‐11		5.04 ± 0.03^bA^	10.77 ± 0.14^cB^	0.42 ± 0.03^cC^	3.44 ± 0.37^abD^	2.38 ± 0.01^bE^
	37x‐6		ND	4.86 ± 0.35^bB^	9.70 ± 0.07^dC^	24.9 ± 3.22^cD^	1.26 ± 0.11^aAB^
	37x‐8		5.19 ± 0.01^cA^	1.86 ± 0.01^aB^	1.68 ± 0.01^eC^	4.69 ± 0.00^bD^	1.57 ± 0.04^aE^
	CO	20	5.45 ± 0.07aA	18.31 ± 0.01^aB^	21.11 ± 0.08^dC^	34.35 ± 0.02^bD^	31.92 ± 0.05^aE^
	x3‐3b		ND	3.34 ± 0.02^bC^	1.68 ± 0.03^aB^	1.46 ± 0.35^aB^	29.37 ± 0.32^bD^
	30x‐11		5.04 ± 0.03bA	22.00 ± 0.04^cB^	21.5 ± 0.71^dB^	44.26 ± 3.08^cC^	25.09 ± 0.11^cB^
	37x‐6		ND	18.5 ± 0.07^aB^	11.78 ± 0.03^bC^	44.40 ± 0.49^cD^	24.41 ± 0.22^dE^
	37x‐8		5.19 ± 0.01^cA^	32.95 ± 2.23^dC^	18.00 ± 1.41^cB^	50.61 ± 0.54^dD^	15.27 ± 0.01^eB^
Putrescine	CO	4	41.18 ± 0.07^aB^	16.51 ± 1.12^aB^	9.65 ± 0.02^aA^	21.84 ± 0.14^cB^	10.37 ± 0.42^cA^
	x3‐3b		5.49 ± 0.09^bA^	4.05 ± 0.05^bB^	0.81 ± 0.01^bC^	6.05 ± 0.31^aD^	6.15 ± 0.02^aE^
	30x‐11		33.38 ± 0.13^cA^	19.76 ± 0.02^cB^	8.27 ± 0.07^cC^	5.19 ± 0.45^aD^	14.10 ± 0.01^dE^
	37x‐6		21.54 ± 0.06^dC^	9.33 ± 0.48^dB^	3.87 ± 0.03^dA^	10.27 ± 1.36^bB^	3.90 ± 0.21^bA^
	37x‐8		30.39 ± 0.10^eA^	18.23 ± 0.01^eB^	6.55 ± 0.03^eC^	6.34 ± 0.02^aD^	4.43 ± 0.01^bE^
	CO	20	41.18 ± 0.07^aA^	67.30 ± 0.39^bB^	111.19 ± 1.35^dC^	155.68 ± 0.38^bD^	159.63 ± 0.01^cE^
	x3‐3b		5.49 ± 0.09^bA^	5.67 ± 0.05^aA^	4.16 ± 0.01^aA^	16.72 ± 1.71^aB^	97.89 ± 0.93^aC^
	30x‐11		33.38 ± 0.13^cA^	76.44 ± 0.00^bB^	109.62 ± 0.83^dC^	174.13 ± 0.59^dD^	190.87 ± 19.33^bE^
	37x‐6		21.54 ± 0.06^dA^	39.88 ± 0.15^cB^	91.15 ± 2.72^bC^	105.67 ± 1.38^cD^	164.17 ± 1.22^dE^
	37x‐8		30.39 ± 0.10^eA^	68.37 ± 1.71^dD^	82.26 ± 0.86^bC^	151.49 ± 1.54^bC^	88.18 ± 0.18^aB^
Cadaverine	CO	4	30.55 ± 0.07^aB^	2.31 ± 0.20^aD^	0.64 ± 0.01^aA^	1.03 ± 0.11^aC^	0.76 ± 0.03^aA^
	x3‐3b		14.75 ± 0.01^bA^	ND	ND	ND	ND
	30x‐11		23.26 ± 0.04^cA^	3.03 ± 0.01^bB^	2.01 ± 0.01^bC^	1.00 ± 0.07^aD^	5.95 ± 0.00^bE^
	37x‐6		20.02 ± 0.01^dA^	4.31 ± 0.19^cB^	2.83 ± 0.00^bC^	ND	2.28 ± 0.07^cD^
	37x‐8		18.13 ± 0.07^eB^	3.69 ± 0.00^dA^	37.33 ± 0.90^cC^	4.12 ± 0.01^bA^	11.67 ± 0.03^dD^
	CO	20	30.55 ± 0.07^aA^	1.77 ± 0.12^aB^	4.11 ± 0.03^aC^	3.86 ± 0.01^bD^	2.64 ± 0.04^aE^
	x3‐3b		14.75 ± 0.01^bB^	36.51 ± 0.04^bC^	0.76 ± 0.03^bA^	0.93 ± 0.27^aA^	2.47 ± 0.58^aD^
	30x‐11		23.26 ± 0.04^cC^	3.53 ± 0.59^aA^	3.73 ± 0.01^cA^	27.46 ± 1.81^cD^	11.81 ± 0.04^bD^
	37x‐6		20.02 ± 0.01^dA^	39.77 ± 0.61^cB^	11.84 ± 0.04^dC^	1.66 ± 0.01^aD^	34.32 ± 0.42^cE^
	37x‐8		18.13 ± 0.07^eA^	25.26 ± 1.60^dB^	49.73 ± 0.04^eC^	13.47 ± 0.13^dD^	55.34 ± 0.12^dE^
Tryptamine	CO	4	2.54 ± 0.08^aA^	1.73 ± 0.08^aB^	ND	ND	0.74 ± 0.01^aC^
	x3‐3b		ND	ND	ND	ND	ND
	30x‐11		3.03 ± 0.04^bA^	2.23 ± 0.02^bB^	ND	ND	0.84 ± 0.01^bC^
	37x‐6		ND	ND	ND	ND	ND
	37x‐8		ND	ND	ND	ND	ND
	CO	20	2.54 ± 0.08^aA^	2.07 ± 0.04^aB^	ND	1.72 ± 0.00^aC^	2.94 ± 0.00^aD^
	x3‐3b		ND	ND	ND	ND	1.65 ± 0.02^bA^
	30x‐11		3.03 ± 0.04^bB^	2.17 ± 0.01^bA^	ND	5.49 ± 0.56^bB^	2.89 ± 0.01^cC^
	37x‐6		ND	ND	ND	ND	4.01 ± 0.01^dA^
	37x‐8		ND	ND	ND	ND	1.93 ± 0.01^eA^
2‐Phenylethylamine	CO	4	8.11 ± 0.07^aA^	ND	ND	ND	ND
	x3‐3b		ND	ND	ND	ND	ND
	30x‐11		5.60 ± 0.04^bA^	1.80 ± 0.01^aB^	1.63 ± 0.06^aB^	ND	4.26 ± 0.02^aC^
	37x‐6		4.93 ± 0.06^cA^	ND	ND	ND	ND
	37x‐8		ND	ND	ND	ND	ND
	CO	20	8.11 ± 0.07^aA^	4.65 ± 0.04^aB^	ND	1.77 ± 0.01^aC^	3.29 ± 0.00^aD^
	x3‐3b		ND	ND	ND	ND	2.32 ± 0.04^bA^
	30x‐11		5.60 ± 0.04^bA^	6.81 ± 0.02^bA^	53.59 ± 2.04^aB^	29.48 ± 1.86^bC^	37.01 ± 0.10^cD^
	37x‐6		4.93 ± 0.06^cA^	3.66 ± 0.01^cB^	ND	ND	2.50 ± 0.04^dC^
	37x‐8		ND	ND	ND	ND	ND
Spermidine	CO	4	3.02 ± 0.01^dD^	2.26 ± 0.21^bcC^	0.23 ± 0.00^aA^	3.67 ± 0.55^cD^	1.11 ± 0.04^aB^
	x3‐3b		2.66 ± 0.14^cC^	2.44 ± 0.01^cC^	1.20 ± 0.03^cA^	1.67 ± 0.13^bB^	1.27 ± 0.23^aA^
	30x‐11		2.00 ± 0.14^bC^	1.72 ± 0.03^abC^	0.90 ± 0.14^bB^	0.58 ± 0.04^aA^	1.02 ± 0.18^aB^
	37x‐6		1.17 ± 0.01^aB^	1.20 ± 0.51^aB^	0.25 ± 0.01^aA^	0.68 ± 0.04^aAB^	1.96 ± 0.32^bC^
	37x‐8		1.89 ± 0.06^bD^	1.35 ± 0.05^aC^	0.82 ± 0.07^bB^	0.51 ± 0.06^aA^	0.89 ± 0.19^aB^
	CO	20	3.02 ± 0.01^dB^	1.47 ± 0.13^aC^	0.78 ± 0.03^bD^	1.75 ± 0.08^bA^	1.78 ± 0.16^bA^
	x3‐3b		2.66 ± 0.14^cB^	3.16 ± 0.43^bB^	2.48 ± 0.07^dB^	1.16 ± 0.62^abA^	0.80 ± 0.03^aA^
	30x‐11		2.00 ± 0.14^bD^	0.96 ± 0.06^aA^	1.31 ± 0.01^cB^	1.64 ± 0.06^bC^	2.05 ± 0.12^bD^
	37x‐6		1.17 ± 0.01^aAB^	1.32 ± 0.59^aB^	0.57 ± 0.10^aA^	0.75 ± 0.09^aAB^	0.90 ± 0.13^aAB^
	37x‐8		1.89 ± 0.06^bB^	1.67 ± 0.69^aB^	0.63 ± 0.00^aA^	1.13 ± 0.09^abAB^	0.61 ± 0.17^aA^
Total biogenic amines	CO	4	232.55 ± 0.62^eD^	167.79 ± 8.1^cC^	89.34 ± 1.45^dA^	231.06 ± 0.91^dD^	109.39 ± 3.76^dB^
	x3‐3b		43.6 ± 0.45^aC^	45.85 ± 0.31^bC^	18.55 ± 1.13^aB^	62.59 ± 1.7^bD^	13.61 ± 1.42^aA^
	30x‐11		149.82 ± 1.42^dB^	193.09 ± 0.14^dC^	124.55 ± 1.44^eA^	123.1 ± 7.21^cA^	153.14 ± 1.15^eB^
	37x‐6		71.19 ± 0.62^bC^	35.58 ± 0.93^aB^	24.67 ± 0.07^bA^	72.93 ± 6.66^bC^	20.35 ± 3.19^bA^
	37x‐8		109.54 ± 1.15^cE^	51.97 ± 0.01^bC^	65.61 ± 0.51^cD^	44.55 ± 0.18^aB^	38.94 ± 1.68^cA^
	CO	20	232.55 ± 0.62^eAB^	241.06 ± 4.26^bB^	216.2 ± 1.56^cA^	405.19 ± 14.37^cC^	428.12 ± 5.86^cD^
	x3‐3b		43.6 ± 0.45^aB^	89.49 ± 0.69^aD^	35.4 ± 0.35^aA^	75.76 ± 4.76^aC^	199.22 ± 0.85^aE^
	30x‐11		149.82 ± 1.42^dA^	225.91 ± 1.14^bB^	328.71 ± 4.21^dC^	568.26 ± 1.67^dE^	433.23 ± 2.68^cD^
	37x‐6		71.19 ± 0.62^bA^	237.26 ± 1.41^bD^	133.87 ± 0.67^bB^	331.49 ± 3.06^bE^	228.3 ± 3.22^bC^
	37x‐8		109.54 ± 1.15^cA^	285.51 ± 13.78^cD^	217.47 ± 0.61^cB^	344.86 ± 1.81^bE^	239.42 ± 11.32^bC^

Note:

All values are given in mean ± *SD*, where *n* = 3. Values followed by the same capital letters (A–E) in a row and lowercase letters (a–e) in a column are not statistically different (*P < *.05). ND: not detected. CO: control without starter culture; 30x‐11: with 30x‐11 *Pentose Staphylococcus*; 37x‐6: with 37x‐6 *Lactobacillus plantarum*; 37x‐8: with 37x‐8 *Staphylococcus pentosae*; x3‐3b: with x3‐3b *Lactobacillus plantarum*.

TYR was quantitatively the most important BA in our study. The primary BA in fermented sausage is TYR, and the concentration range is 8.02–229.88 mg/kg during the storage. The range of TYR content for fermented sausages was shown as 6.9–267.1 mg/kg in the literature, and it was similar with our results (Roseiro, Santos, & Sol, 2006). As TYR was the most abundant BA in all fermented sausages. Compared with other BAs, TYR may be more likely to cause adverse health effects (Aymerich et al., 2000). In the whole storage process, although the content of TYR in fermented sausages of each group stored at 4°C fluctuated frequently, compared with the beginning and the end of storage, which showed a downward trend, the content of TYR in each group increased during storage at 20°C. Therefore, the content of tyramine in fermented sausages showed a downward trend when stored at 4°C, when the temperature was high, the accumulation of TYR in sausages was more, which was consistent with the research results of Ruizcapillas and Jiménezcolmenero (2004).

HIM is the most common and toxic BA in fermented food (Santos, 1996; Shalaby, 1996). HIM contents of fermented sausage samples at 4°C were in the interval of 0–26.28 mg/kg, which were below toxic levels, and HIM contents of fermented sausage samples at 20°C were in the interval of 1.46–50.61 mg/kg. Compared with room temperature, low temperature of storage can effectively inhibit the growth, reproduction, and metabolism of bacteria and thus reduce the increase in histamine content, which is the same as the research results of Kanki, Yoda, Tsukamoto, and Baba (2007). In addition to the control group, the content of histamine in the four groups of sausages with the addition of starter cultures decreased at 4°C, while the content of histamine in the sausages stored at 20°C increased compared with the content of histamine in the sausages stored for 0 days, which showed that these starter cultures can better limit the content of histamine under the low‐temperature condition.

Very high positive correlations (*r* = .883, *p* < .05) were found between content of total BAs and PUT. Throughout the whole storage process, it can be seen intuitively from the figure that the content of PUT in sausage stored at room temperature was always higher than that in cold storage for all samples. It can be seen that the storage temperatures have great influence on the content of PUT. It is possible that the growth and reproduction of most microorganisms are limited, their metabolic capacity is reduced, and the accumulation of PUT is relatively reduced during low‐temperature storage. This is due to the slow metabolism of bacteria and low enzyme activity at low temperature, so the yield of amines is very small (Kanki, Yoda, Tsukamoto, & Baba, 2007).

As the result of PUT, the content of CAD in fermented sausage of all group stored at 20°C was significantly higher than that stored at 4°C. In general, low temperature leads to low capacity of amine production, and with the extension of storage time, the content of CAD in low‐temperature storage showed a downward trend, while in room temperature storage, CAD rises repeatedly. The content of PHE and TRY in fermented sausages was relatively low under low storage temperature. SPD is one of the most common BAs in raw meat. In this experiment, SPD exists in every storage stage, and the content of SPD was always between 0.23 and 3.67 mg/kg in different groups changes little during storage. This is consistent with the results of Komprda et al. (2001), the content of SPD has no significant relationship with the added starter, fermentation time, fermentation temperature, and storage time, and the content of SPD has remained relatively stable in a series of processing and storage processes.

## 4 CONCLUSION

In our study, different amounts of BAs can be produced in different groups under the influence of four starter cultures. In terms of decarboxylase activity, x3‐3b, 37x‐6, and 37x‐8 are declared negative and 30x‐11 was positive. The content of BAs with 37x‐6, x3‐3b, and 37x‐8 was obviously decreased at 4°C storage, the content of TRM, PUT, CAD, and HIM of all samples storage at 20°C was higher than that storage at 4°C after 42 days. The effect of temperature was significant (*p* < .05) for BAs tested during the storage. In addition, the pH value of the treatment groups was significantly below the content of sample CO (*p* < .05), the a* value of the treatment groups was greater than that of sample CO (*p* < .05), but the water activity value, L* and b*of each group, had no significant difference (*p* > .05). The producer should select appropriately the starter cultures to reduce the content of BA in fermented sausages. Consumers should store fermented sausages in refrigerated conditions. Products stored at room temperature may produce more BAs. It is suggested that legislators should establish the limit of BA content in fermented sausage.

## CONFLICTS OF INTEREST

All coauthors declare that they have no conflict of interest.

## ETHICAL APPROVAL

This study does not involve any human or animal testing.
